# Super-Resolution Microscopy: A Virus’ Eye View of the Cell

**DOI:** 10.3390/v6031365

**Published:** 2014-03-19

**Authors:** Joe Grove

**Affiliations:** Institute of Immunity and Transplantation, University College London, London NW3 2PF, UK; E-Mail: j.grove@ucl.ac.uk; Tel.: +44-20-7794-0500 (ext. 22472)

**Keywords:** microscopy, super-resolution, PALM, STORM, dSTORM, tetraspanin, virus assembly, virus entry

## Abstract

It is difficult to observe the molecular choreography between viruses and host cell components, as they exist on a spatial scale beyond the reach of conventional microscopy. However, novel super-resolution microscopy techniques have cast aside technical limitations to reveal a nanoscale view of virus replication and cell biology. This article provides an introduction to super-resolution imaging; in particular, localisation microscopy, and explores the application of such technologies to the study of viruses and tetraspanins, the topic of this special issue.

## 1. Introduction

The nanoscopic world of virology and molecular cell biology is barely within reach of comprehension: the size difference between a virus (~100 nm) and a human being (~1.7 m) is comparable to that between an apple and the United Kingdom or, for the benefit of readers in the U.S., the state of Wyoming. This presents not only a problem of comprehension but, more importantly, a technical barrier; how do we observe processes occurring at this scale?

The fluorescence microscope is the principal tool for investigating the spatial organisation of bio-molecular processes. However, due to the inherent properties of light, optical resolution is limited by diffraction to ~1/2 the wavelength of fluorescent light. Practically, this means that objects smaller than 200–300 nm cannot be accurately resolved. Various techniques have been developed to over-come this limit. Electron microscopy (EM) exploits the incredibly short wavelength of electron beams to achieve resolutions up to the subnanometer level [1]; however, sample preparation is onerous and precludes live imaging, and there is only limited opportunity for molecular identification. Single molecule tracking (SMT), discussed in detail elsewhere in this issue, involves sparsely labelling bio-components with fluorescent dyes allowing individual molecules to be tracked in live cells with a precision of 1–50 nm [2,3]. Although SMT yields invaluable observations of molecular dynamics, investigations are limited to a small sub-set of molecules such that sub-cellular structures cannot be observed and tracking multiple species in the same sample remains challenging. 

Recent advances in photochemistry, optical engineering and data analysis have led to the development of a number of alternative fluorescence microscopy techniques that circumvent the diffraction limit. This, so called, super-resolution microscopy has opened the door to true nanoscale mapping of biological components, providing an unprecedented view of virus replication and its underlying cell biology.

## 2. Super-Resolution Imaging

**Figure 1:**
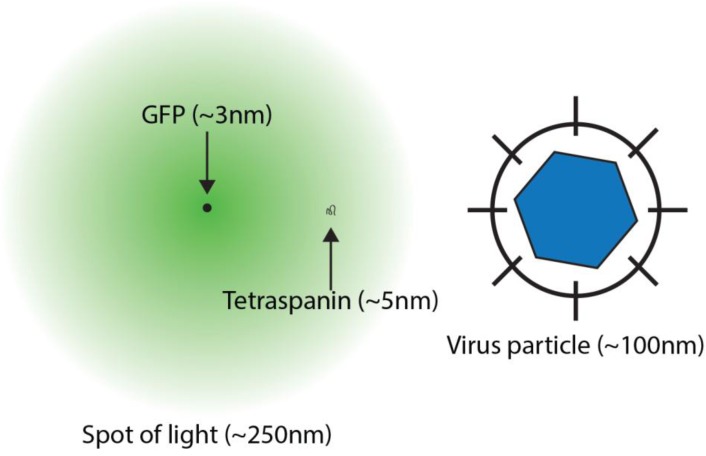
Molecular Scales and Microscopy. The cartoon illustrates the relative sizes of relevant nanoscale objects. Green fluorescent protein (GFP) has a roughly cylindrical structure measuring ~2 × 5 nm, however, when viewed under a microscope the light emitted from a single GFP molecule diffracts to appear as a spot of light measuring approximately 250 nm in diameter. The large extracellular loop of a tetraspanin extends for 3–5 nm, whereas virus particles typically range from 20–1000 nm.

As first analytically described by Ernst Abbe in 1873, the maximum resolution of a microscope is limited by the intrinsic diffraction of light as it passes through the optical components, on its journey from the sample to the observer [4]. The consequence of this property is that light travelling from a miniscule point source, such as an individual molecule of green fluorescent protein (GFP), will spread out to appear as a spot with a diameter of ~250 nm (Figure 1). In a typical biological sample many thousands of crowded proteins are labelled; the signals from each of these point sources spread out and merge, obscuring the molecular details of the sample. This phenomenon becomes particularly problematic when studying nanoscale macromolecular complexes, for example membrane domains or assembling virus particles. 

In broad terms super-resolution microscopy techniques bypass the conventional resolution limit by reducing the extensive merging of signals from neighbouring fluorophores. Localisation microscopy achieves this by temporally separating the emissions from crowded fluorophores, whereas other techniques spatially modulate fluorescence. For example stimulated emission depletion (STED) microscopy is a confocal technique that uses a depletion laser to turn “off” fluorescent emission in the area surrounding a discrete spot of 10–100 nm, allowing proteins within this spot to be examined in isolation [5]. In structured illumination microscopy (SIM) patterns of light, such as grids or stripes, are projected on to the sample. By acquiring sequential images in which the orientation of these patterns has been changed, mathematical transformations can be used to extract information from below the diffraction limit, increasing the resolution to ~125 nm [6]. For more information on STED and SIM please see Schermelleh *et al*. [7].

## 3. Localisation Microscopy

Unlike other super-resolution modalities, localisation microscopy is capable of individually imaging each of the thousands of fluorophores that decorate a biological sample. The various incarnations of localisation microscopy differ in their underlying photochemistry and analytical approaches, nevertheless they share the same basic principle; fluorophores are induced to transiently blink “off” and “on” such that very few emit light simultaneously, allowing individual probes to be located with a high precision [8,9,10]. 

**Figure 2:**
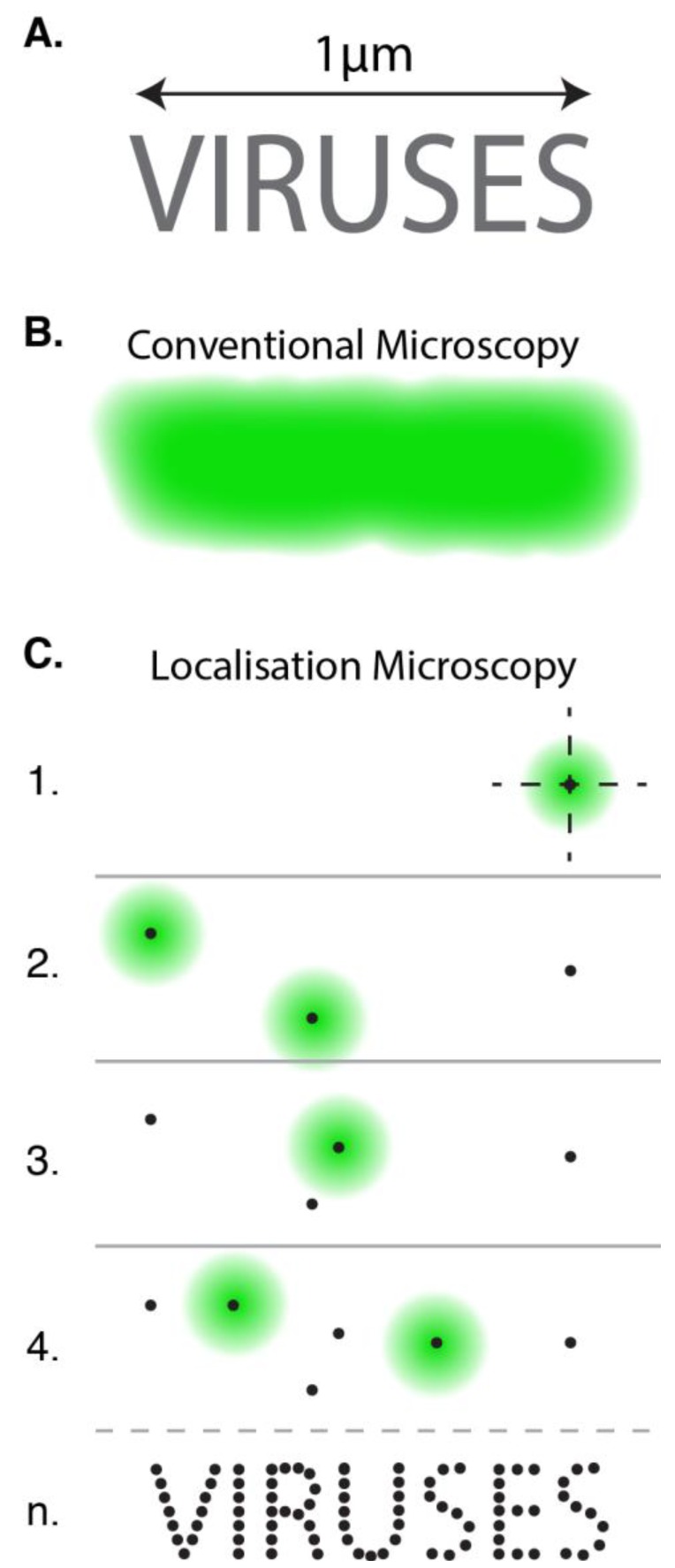
Localisation Microscopy. (**A**, **B**) The ultrastructure of a fluorescently labeled nanoscale object cannot be discerned by conventional microscopy as the signals from apposing fluorophores merge together. (**C**) In localisation microscopy fluorophores are induced to blink “off” and “on” such that their signals do not appear simultaneously. By locating the central position of each individual spot of light and summing all positions over a series of sequential images (n) it is possible to reconstruct the ultrastructure of the object.

To illustrate how this works consider a densely labelled nano-object (Figure 2A). When viewed by conventional fluorescence microscopy the signals from each of the fluorophores merge together and the fine ultrastructure is lost (Figure 2B). In localisation microscopy, various photochemical tricks are used to turn the vast majority of fluorophores “off”, allowing only a small random sub-set to blink “on” at any given moment, a process known as photo-switching. The signals from these sparse molecules appear as individual diffraction limited spots (Figure 2C). The intensity of light across these spots follows a two dimensional Gaussian distribution, by fitting this curve and finding its central peak it is possible to calculate the location of the fluorophore with a very high precision [11]. The ultra-structure of the nanoscale object can subsequently be reconstructed by locating many different fluorophores over time (Figure 2C). Each photon emitted from a photo-switching event represents an independent measurement of the fluorophore’s true position; consequently, the intensity of light (*i.e.*, the number of photons) within a diffraction-limited spot determines the precision of localisation. Typical localisation precisions range from 1–50 nm [11].

Since the inception of localisation microscopy the field has grown to become a jungle of acronyms, each relating details of how fluorophores are made to blink or the exact manner of their localisation. For the purposes of brevity the following section summarises the two principal methods used to achieve photo-switching. 

PALM (PhotoActivated Localization Microscopy) type methods use switchable fluorescent proteins such as PA-GFP (photo-activatable GFP) or mEOS [9,10]. Upon illumination with a short wavelength activation laser, these genetically encoded tags can be triggered to irreversibly transition from a dark “off” state to a fluorescent “on” state. By irradiating the sample with very low intensity activation laser only a small number of switchable proteins will transition to the “on” state at any given moment. Simultaneous illumination with a high intensity excitation laser ensures that activated proteins emit a bright fluorescent signal before rapidly photobleaching to a dark state. Consequently the signals from each probe appear randomly and transiently such that molecular localisation can be performed. One particular benefit of this method is that each probe has a high probability of appearing only once, allowing estimates of molecular stoichiometry [12,13,14]. 

STORM (STochastic Optical Reconstruction Microscopy) type methods employ standard organic dyes that can be driven into a metastable “off” state in the presence of imaging buffer containing oxygen scavenging enzymes and/or reducing agents. Stochastic recovery to the fluorescent “on” state is then achieved either by coupling to a secondary activator dye (in the original STORM methodology) [8] or by relying on the intrinsic cycling of fluorophores between “on” and “off” states (in direct STORM, *i.e.*, dSTORM) [15]. 

An understanding of the precise chemistry of fluorophore blinking is not essential for the budding localisation microscopist. However, optimal imaging often requires sample-specific adjustment of photochemical parameters, such as laser power or buffer constituents. This becomes critical when studying discrete nanoscale objects, such as virus particles, as it is necessary to accumulate sufficient localisations to describe the entire structure. For example, the individual characters of the nanoscale text in Figure 2 only become apparent once an adequate number of localisations are collected. This concept is known as the Nyquist sampling theorem [16] and is an important factor in determining the practical resolution of localisation microscopy. For detailed discussion of the technical aspects of photo-switching, Nyquist sampling and resolution, readers are directed to these excellent reviews [17,18,19]. There are also a number of articles giving step-by-step protocols to perform localisation microscopy techniques [20,21,22,23]. 

As a nascent technology, localisation microscopy drew two major criticisms. Accumulation of sufficient data points to reconstruct an image is limited by the efficiency and rate of photo-switching. Consequently a single super-resolution image or time point typically requires acquisition for 5–30 min, precluding the opportunity for live-cell molecular imaging. Additionally, initial implementations were unable to locate molecules in the axial (Z) dimension and/or perform optical sectioning; therefore early studies were performed under TIRF illumination, restricting observations to within ~100 nm of the glass coverslip. However, innovations in signal detection and particle localisation have recently removed these limitations, allowing live cell video-rate localisation microscopy and 3D imaging with an axial resolution of 10–100 nm, albeit in the hands of very experienced microscopists [24,25,26].

Nonetheless, even when limited to TIRF imaging of fixed samples, localisation microscopy is an exceptional tool for investigating the molecular organisation of the plasma membrane, making it particularly well suited to studying processes such as virus entry and assembly, and addressing fundamental questions regarding tetraspanin biology.

## 4. Super-Resolved Viruses

As yet, only a handful of reports have applied super-resolution imaging to study the biology of viruses, and unsurprisingly, given the wealth of pre-existing tools, the principal focus has been HIV replication. A variety of labelling strategies have allowed molecular imaging of HIV structural components with sufficiently high resolution to distinguish the conical morphology of the mature capsid and the internal architecture of virions, including the encapsidated integrase enzyme and peripheral envelope glycoproteins (Env) [27,28]. Beyond these impressive technical feats, initial studies have concentrated on re-evaluating the fundamental processes of particle assembly and virus entry, offering novel insights into these well-trodden topics. 

Dynamic analysis of the major HIV structural protein, Gag, by sptPALM (a technique that combines localisation microscopy and SMT) suggests a diffusing population of membrane-associated Gag that are recruited and confined to discrete assembly clusters [29]. Independent studies of fixed cells using PALM/STORM defined these as roughly spherical assembly sites of ~130 nm, consistent with accounts of the size of mature HIV particles [28,30,31]. Two-colour localisation microscopy revealed recruitment of Env in a ring like formation around assembly sites; this association was dependent both on the intracellular tail of Env and the matrix domain of Gag [31,32]. 

**Figure 3:**
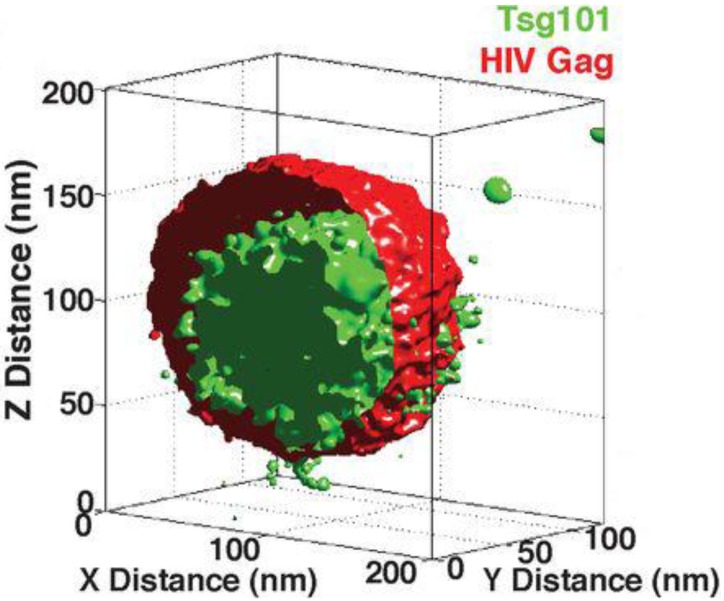
The Molecular Architecture of HIV. An averaged 3D probability density map displaying the distribution of Gag and TSG101 in nascent HIV virus like particles, adapted from Distribution of ESCRT Machinery at HIV Assembly Sites Reveals Virus Scaffolding of ESCRT Subunits [33]. Reprinted with permission from AAAS.

The release of HIV particles from the cell surface is mediated by the membrane fission activity of the host ESCRT (endosomal sorting complex required for transport) machinery. By combining 3D localisation microscopy and particle averaging van Engelenburg *et al*. recently demonstrated Gag dependent recruitment of ESCRT components to the inside of nascent virus-like particles [33]. This suggests that the ESCRT apparatus orchestrates membrane fission from within virions. Figure 3 is taken from this study and displays virion architecture as an averaged 3D probability map of Gag and TSG101, an ESCRT-I component.

Following budding, HIV undergoes proteolytic maturation to an entry competent particle. Chojnaki *et al*. used STED microscopy to demonstrate capping of Env at the virion surface concomitant with particle maturation, suggesting that virion architecture is primed for engagement with successive target cells [34]. Finally, nanoscopic measurements of isolated particles undergoing virus entry and nuclear import reveal sequential shifts in particle size suggesting a series of structural rearrangements associated with virus ingress [27,35]. 

These examples, although limited in number, illustrate the potential of super-resolution imaging to provide a nanoscopic window on cell-biological processes. Many fields will benefit from this molecular clarity, arguably none more so than the study of tetraspanins.

## 5. Observing Tetraspanin Biology

Tetraspanins are integral transmembrane proteins found principally at the plasma membrane, however, they remain a resolutely enigmatic group of proteins, leaving them primarily defined by structure and biochemistry. They consist of two extracellular loops anchored by four transmembrane domains, with conserved cysteine residues necessary both for di-sulphide bridges within the large extracellular loop and extensive palmitoylation of intracellular domains [36]. 

Largely without native ligands, tetraspanins exert control over plasma membrane biology via interactions with various binding partners. However, it is not necessarily the discrete partner-pair interactions of individual tetraspanins that confers function but more their ability to assemble higher-order complexes, referred to tetraspanin enriched microdomains (TEMs). In this way, tetraspanins act to functionally compartmentalise plasma membrane activities, for example tetraspanins co-ordinate the distribution of various integrins to regulate adhesion (in the case of CD151) and sperm-egg fusion during fertilisation (by CD9 and CD81) [36]. It is likely that this molecular scaffolding property is what makes them attractive targets for viruses. 

Although biochemical analysis, immunoprecipitation and genetic studies have identified a wealth of tetraspanin binding partners and associated functions, these techniques provide scant information on the spatial distribution of membrane components. It is therefore unsurprising that the precise nature of TEMs have remained elusive; for instance the size, distribution and internal architecture of these membrane domains is largely unknown. However, imaging studies using various diffraction limited microscopy techniques, EM and SMT have shed light on TEMs [3,37,38] and, in particular, their role in virus replication. 

A number of reports have combined quantitative fluorescence microscopy and immunogold electron microscopy to describe TEMs and their role in HIV replication. Nydegger *et al*. observed TEMs as discrete plasma membrane domains of ~400 nm in diameter, composed of variable combinations of tetraspanins and, presumably, their binding partners. In HIV infected cells virus assembly is targeted to these domains, suggesting that they act as scaffolds for viral organisation [39]. Moreover, Gag is capable of clustering tetraspanins and inducing coalescence of TEMs with lipid rafts [40,41]. 

Imaging approaches have also been used to tackle the role of tetraspanins in virus entry. Harris *et al*. used FRET microscopy to address the role of CD81 in hepatitis C virus (HCV) ingress. Although FRET imaging is not a super-resolution technique per se, it exploits the transfer of energy between closely apposing fluorophores to make sub-diffraction limit measurements of the distances between proteins. In a series of studies the authors used FRET to demonstrate that direct interaction between CD81 and claudin-1 is necessary for efficient HCV infection [42,43,44]. In further support of the notion that tetraspanin biology is determined by interactions with binding partners, Potel *et al*. used SMT to demonstrate that immobilisation of CD81 via interaction with EWI-2wint abrogates HCV infection [45]. The use of SMT strategies to study the dynamics of tetraspanins and their role in virus replication is covered in detail elsewhere in this issue, therefore readers are directed to this and other articles [3,46,47]. 

Although these various microscopy techniques allow quantitative and dynamic observation of the cell surface, they provide limited information on the nanoscale compartmentalisation of the plasma membrane. This remains a critical gap in our knowledge, especially given that the function of tetraspanins is defined by their organisation into discrete membrane domains alongside specific binding partners. However, the advent of localisation microscopy represents a unique opportunity to observe the composition and architecture of TEMs. 

The Zhaung group, one of the pioneering labs in localisation microscopy, recently combined conventional microscopy, EM and STORM to evaluate the role of CD81 in Influenza A virus (IAV) replication [48]. Basic virological assays indicated that CD81 plays a role both in virus entry and release. By following fluorescently labelled virus particles it was revealed that IAV frequently penetrates from within CD81 positive maturing endosomes and in the absence of CD81 fusion was impaired. Following entry and replication, IAV particles assemble at the cell surface. Immunogold EM analysis indicated that CD81 is enriched both at the tip and neck of nascent budding virions. Upon depletion of CD81, whilst particle assembly was unperturbed, the release of mature virions from the cell surface was defective, suggesting a role for tetraspanins in scission of the IAV particle envelope. Interestingly, these roles for CD81 in entry and release both suggest a link between tetraspanins and membrane manipulation by IAV. Finally, the authors used STORM localisation microscopy to analyse the 3D distribution of CD81 along filamentous IAV particles, achieving a lateral resolution (xy) of ~20 nm and ~50 nm axially (z). Surprisingly, CD81 is arranged in evenly distributed puncta (~150 nm apart) along the entire length of the particle, in an alternating pattern to the IAV protein PB1. However, it is unknown whether this distribution indicates a further role for CD81 in the function and morphology of mature IAV particles.

Thus far the most extensive use of localisation microscopy to describe TEM function does not concern virus replication. Mattila *et al*. used dSTORM and SMT to evaluate the molecular coalescence of the B-cell receptor (BCR) and co-factor CD19 upon primary B-cell activation [49]. They found that CD81 determined the mobility and cluster density of CD19, controlling its availability to the BCR and regulating B-cell activation. They propose a model in which CD19 is immobilised in TEMs such that it is partitioned from the BCR but poised for activation. Following stimulus actin remodelling allows the BCR to access TEM resident CD19, facilitating efficient signal transduction. 

This elegant study exploits a unique feature of localisation microscopy, the potential to count and map individual molecules. Raw localisation data consists of a list of Cartesian coordinates that describe the position of each molecule in three-dimensional space. Such point co-ordinates are invaluable when considering the distribution of biological components. A wealth of spatial statistics tools, previously employed to analyse macroscale problems such as the geographical distribution of plants, can now be applied to nanoscale phenomena [50,51,52]. Mattila *et al*. demonstrate the power of these approaches by identifying subtle changes in the molecular distribution of receptor that are barely detectable by human scrutiny of the corresponding images. 

## 6. A Virus’ Eye View of CD81

**Figure 4:**
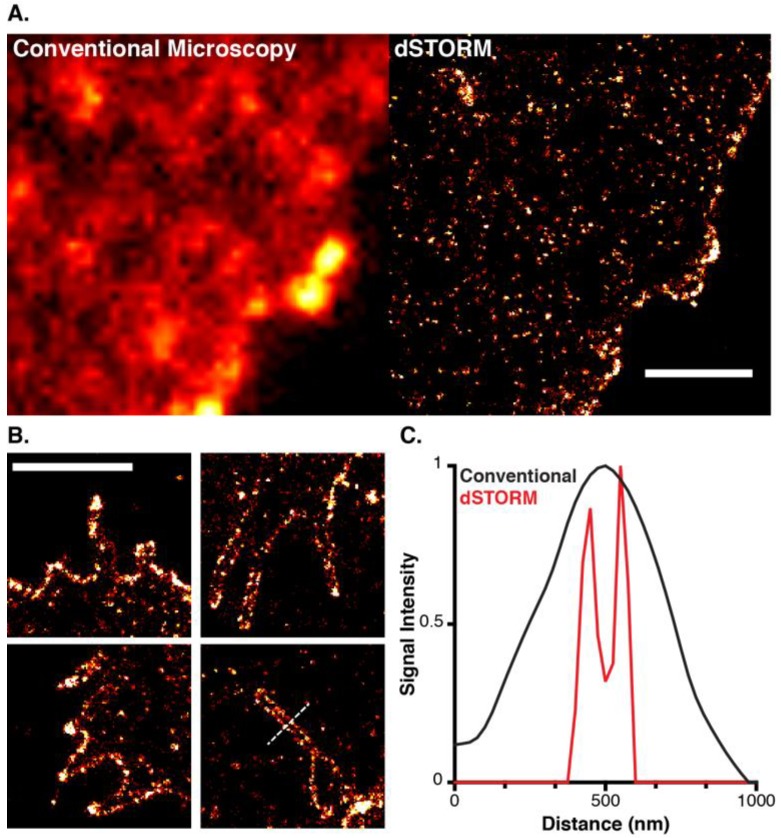
A Virus’ Eye View of CD81. (**A**) Fixed human hepatoma Huh-7.5 cells labeled with Alexa Fluor 647 conjugated anti-CD81 imaged by conventional TIRF microscopy and dSTORM, as described in Metcalf *et al*. [21]; (**B**) Enrichment of CD81 on membrane tubules, most likely microvilli; (**C**) A line profile taken through the membrane structure indicated in lower right (**B**), the lumen of the tubule is clearly discernable in the dSTORM plot (red), whereas the corresponding conventional microscopy plot (black) is relatively featureless. Scale bars, 2 µm.

To fully illustrate the improvement in resolution afforded by localisation microscopy Figure 4 displays the cell surface distribution of antibody labelled CD81 by diffraction limited TIRF microscopy and super-resolved dSTORM imaging [15,21,53]. Whereas, the standard fluorescence micrograph appears relatively featureless, the reconstructed dSTORM image reveals the organisation of CD81 into distinct puncta of varying size and intensity that may represent individual TEM. CD81 was also frequently enriched on membrane protrusions, possibly microvilli (Figure 4B). This is particularly notable given the implication of tetraspanins in virus particle budding [39,48], and may reflect a general role for CD81 in membrane sculpture. As a further demonstration of the resolution achieved by localisation microscopy, the ~100 nm lumen of the membrane structures is clearly discernable (Figure 4C).

## 7. Conclusions and Perspectives

Super-resolution microscopy is a very powerful technique that is set to change the way in which we view cells, organelles, viruses and their various molecular components. The ability of localisation microscopy to map individual proteins and its suitability for studying the plasma membrane make it an ideal technique for dissecting virus entry and assembly, tetraspanin organisation and function, and the various intersections of these topics. Relatively obvious, and yet not trivial questions, include how the organisation and composition of TEMs regulate HIV assembly sites [39], and whether TEMs represent preassembled entry complexes for pathogens such as HCV or papillomavirus [44,54]. As demonstrated in Figure 4B, localisation microscopy may also offer a new understanding of the ability of tetraspanins to associate with and/or regulate curved membrane structures such as microvilli, exosomes and nascent virus particles [39,48,55,56,57].

A benefit of localisation microscopy not yet covered in this article is its relative affordability. Although a number of turnkey commercial systems have recently become available, the majority of localisation microscopy is still performed on cost-effective “home-built” platforms. These range from cutting-edge developmental systems to a microscope built from inexpensive component parts mounted on a simple “breadboard” stage [58]. This affordability, combined with a wealth of open-source image reconstruction software [53,59,60,61], make localisation microscopy a surprisingly accessible technology. I urge readers to find, buy or, better still, build a microscope and gain a nanoscopic view of the realm of viruses and tetraspanins. 
